# *Neisseria gonorrhoeae* antimicrobial susceptibility testing: application of traditional agar dilution to a high-throughput 96-well microtiter assay

**DOI:** 10.1128/spectrum.03249-24

**Published:** 2025-06-09

**Authors:** Joyce E. Kuipers, Laura Seidel, Nicole E. Garnier, Cezar M. Khursigara

**Affiliations:** 1 Department of Molecular and Cellular Biology, College of Biological Science, University of Guelph, Guelph, Ontario, Canada; Taichung Veterans General Hospital, Taichung, Taiwan, Province of China

**Keywords:** antimicrobial resistance, *Neisseria gonorrhoeae*, susceptibility testing

## Abstract

The global rise in *Neisseria gonorrhoeae* infections and increasing antimicrobial resistance underscores the urgent need for accessible and efficient antimicrobial susceptibility testing methods. While accurate, the gold standard agar dilution method is labor-intensive and resource-demanding, limiting its practicality for research. To address this, we developed and validated a high-throughput 96-well microtiter assay as a viable alternative for measuring minimum inhibitory concentrations (MIC). In addition, we assessed the use of the simple-defined Kellogg’s supplement compared to the CLSI-recommended complex-defined growth supplement. Using 17 standard laboratory strains with different resistance profiles, we evaluated the assay against azithromycin, ceftriaxone, ciprofloxacin, and gentamicin. Both 96-well microtiter assays demonstrated no major errors for any antimicrobial, with some minor errors with azithromycin and consistent minor errors with gentamicin. Essential agreement (EA) and categorical agreement (CA) ranged from 33% to 100%, with gentamicin contributing the most variability across methods. This study establishes the 96-well microtiter assay with Kellogg’s supplement as a reliable and scalable tool for *N. gonorrhoeae* antimicrobial susceptibility testing, reducing the time and resources required for resistance profiling.

**IMPORTANCE:**

The rapid emergence of antimicrobial resistance in *Neisseria gonorrhoeae* presents a critical challenge for global public health. Accurate and accessible methods for assessing antimicrobial susceptibility are essential for monitoring resistance trends and guiding effective treatment strategies. However, traditional methods, such as agar dilution, are labor-intensive and resource-intensive, limiting their widespread application in research. Our study introduces a 96-well microtiter assay that simplifies and scales up susceptibility testing while maintaining the accuracy of the gold standard. By validating the use of the simpler alternative, Kellogg’s supplement, we further lower the barriers to adoption in research settings. This innovation supports timely and efficient antimicrobial resistance surveillance and enhances the capacity for high-throughput testing of novel therapeutic compounds. By increasing the feasibility of susceptibility testing, this method can potentially improve response strategies against drug-resistant gonorrhea and other fastidious pathogens.

## INTRODUCTION

*Neisseria gonorrhoeae* is the causative agent of gonorrhea, a sexually transmitted infection of significant public health concern. In 2017, the World Health Organization (WHO) listed *N. gonorrhoeae* as a high-priority pathogen for antimicrobial research due to the rising prevalence of multidrug-resistant isolates worldwide (1). This trend has spurred renewed efforts to develop novel antimicrobials and alternative therapeutics (2–5). However, as the Centers for Disease Control (CDC) describes, increased reliance on molecular testing rather than culture-based methods has decreased the detection of antimicrobial-resistant isolates (6). This shift is partly due to the challenges of culturing the pathogen and the labor-intensive nature of current susceptibility testing methods. Additional complexities arise from differences in result interpretation between two major reference organizations: the Clinical and Laboratory Standards Institute (CLSI) and the European Committee on Antimicrobial Susceptibility Testing (EUCAST). Furthermore, no broth-based media have been validated for clinical testing of *N. gonorrhoeae* (7–11). Minimum inhibitory concentrations (MICs) are typically determined using breakpoint antimicrobial concentrations via agar dilution, disc diffusion, or gradient E-test, with guidelines provided by CLSI and EUCAST.

These methods effectively determine sensitivity categories but often lack the precision required for research or surveillance settings, where consistent and accurate MIC measurements are critical. While disc diffusion and E-test strip gradients are commonly used in clinical and research settings (12, 13), they can produce variable results. For example, E-tests have shown higher MICs for azithromycin and lower MICs for cefixime compared to agar dilution (14–16). As defined by CLSI and EUCAST, agar dilution testing remains the gold standard for resolving discrepant susceptibility results, such as intermediate zones of inhibition for cephalosporins (17, 18).

However, CLSI and EUCAST differ in their recommended methods and criteria for interpreting categorical breakpoints. For example, CLSI provides disc diffusion breakpoints, while EUCAST does not. In addition, EUCAST guidelines are more stringent for ceftriaxone and ciprofloxacin. Ciprofloxacin is considered resistant at MICs of ≥0.06 µg/mL by EUCAST but at ≥1 µg/mL by CLSI. Similarly, ceftriaxone is resistant at ≥0.125 µg/mL according to EUCAST but at >0.25 µg/mL under CLSI. These differences can complicate the choice of testing methods for a laboratory and may potentially restrict the capabilities of labs focused on antimicrobial discovery, depending on the selected method. Another challenge is the absence of interpretative criteria for gentamicin, an alternative therapeutic for extragenital infections, as recognized by the CDC and WHO (19–21). As *N. gonorrhoeae* continues to develop resistance, consistent monitoring of MICs is essential to detect emerging resistance trends. In addition, access to the gold standard method of MIC testing is crucial for research laboratories dedicated to developing new AMR molecular detection assays.

**Fig 1:**
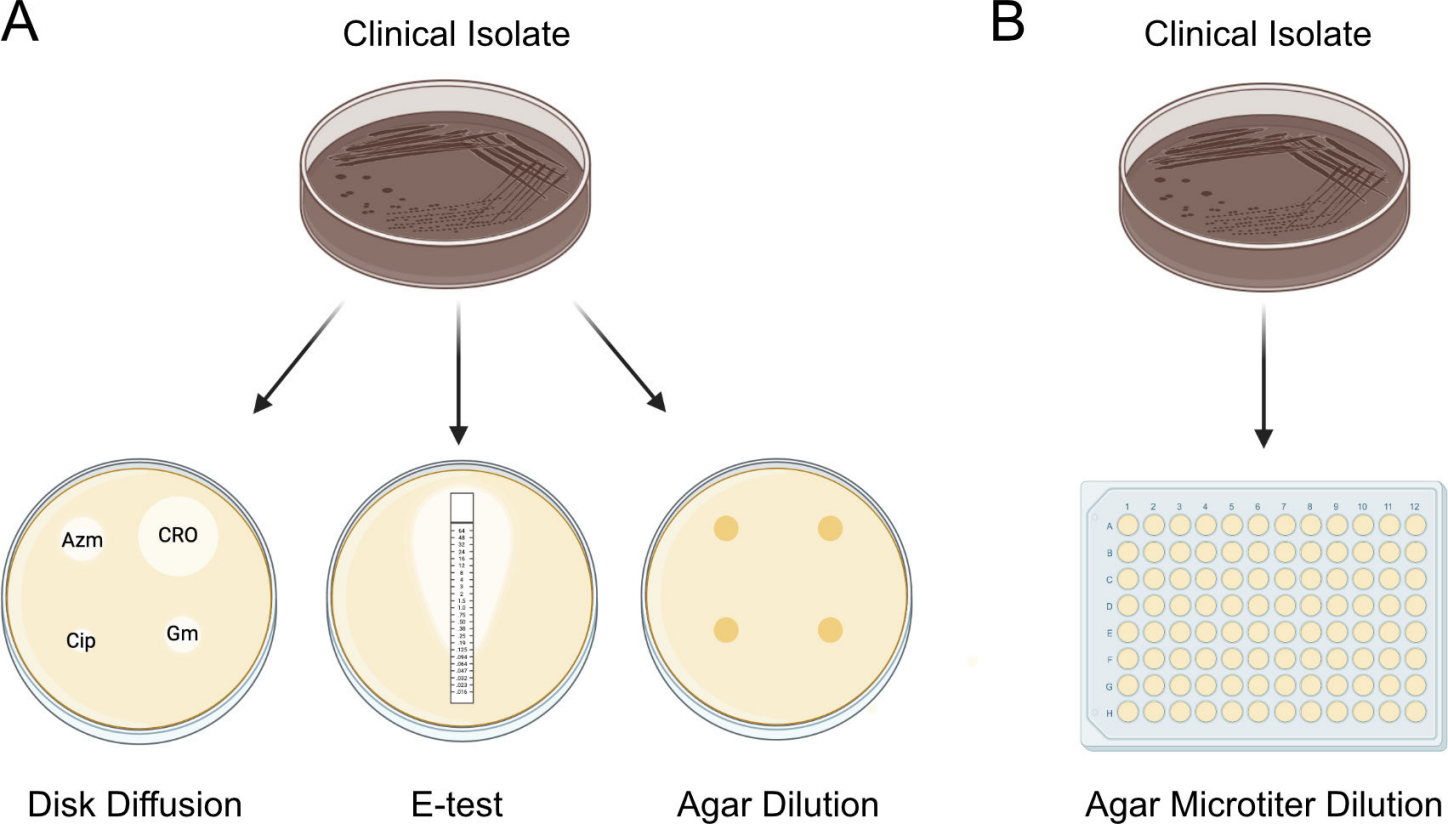
Methods used for determining susceptibility status of *N. gonorrhoeae*. (A) Current methods for determining the susceptibility category and MIC of *N. gonorrhoeae* include disc diffusion, E-test, and agar dilution (the gold standard reference method). (B) The proposed method adapts conventional agar dilution to a high-throughput 96-well microtiter assay. This study also compares the complex-defined growth supplement, recommended by CLSI, with the simple-defined Kellogg’s supplement in the microtiter assay. Abbreviations: Azm (azithromycin), CRO (ceftriaxone), Cip (ciprofloxacin), Gm (gentamicin).

This study presents a novel application of the agar dilution reference method that reduces labor intensity, particularly in the research setting, enabling efficient testing of broader antimicrobial concentration ranges for breakpoint assessment and MIC determination. The technique was developed with the primary objective of establishing and validating a rapid, robust experimental platform for research laboratories. Leveraging the principles of traditional agar dilution, we adapted the process to a 96-well microtiter plate format to validate a microtiter dilution assay (Fig. 1).

We hypothesized that no significant differences would arise between the gold standard method and the microtiter assay results, as the testing media remained unchanged, minimizing the likelihood of variability in MIC values. Our findings supported this hypothesis, with no significant differences between the two methods. However, the antimicrobial gentamicin exhibited the highest variability in categorical agreement (CA), essential agreement (EA), and minor error rates, likely due to tested isolates’ MICs falling within two dilutions of the sensitive and intermediate categories, a known source of variability in testing.

An additional focus was the supplementation required for *N. gonorrhoeae* growth and susceptibility testing. Two widely used supplements for general culturing practices include Kellogg’s and the complex-defined growth supplement (IsoVitaleX) (22–25). While current CLSI and EUCAST guidelines recommend the more expensive and complex-defined growth supplement, we investigated whether it could be replaced with the simpler Kellogg’s supplement. Our results demonstrated that Kellogg’s supplement performed comparably to the defined growth supplement. Thus, a 96-well microtiter dilution assay using Kellogg’s supplement is a practical tool for assessing *N. gonorrhoeae* antimicrobial sensitivities.

## MATERIALS AND METHODS

### *Neisseria gonorrhoeae* growth protocol

In all, 17 strains were used in this study, including ATCC 49226, the standard quality control strain recommended by both CLSI and EUCAST, which was used as a control to assess the validity of each experiment and ensure that appropriate antimicrobial concentrations were achieved. The other strains studied were FA1090, F62, and CH811, generously gifted from the Jo-Anne Dillon laboratory and the 2016 WHO panel, generously gifted from the National Microbiology Laboratory (26–28). Cultures were grown on chocolate agar supplemented with 1% Kellogg’s supplement from glycerol stocks held at −80°C. These cultures were incubated at 37°C in 5% CO2 with 95% humidity. A second passage to supplemented chocolate agar was conducted and incubated for 18–24 hours for antimicrobial susceptibility testing.

### Antimicrobial agar microtiter assay

Antimicrobials used for testing were as follows: ceftriaxone (0.004–2 µg/mL), azithromycin (0.0625–32 µg/mL), gentamicin (0.25–128 µg/mL), and ciprofloxacin (0.125–64 µg/mL) (Sigma Aldrich) (File S1). Extended range testing for strains outside these ranges was as follows: ceftriaxone (0.001–0.002 µg/mL and 4–8 µg/mL), ciprofloxacin (0.001–0.0625 µg/mL), and azithromycin (64–512 µg/mL). To prepare stocks, ciprofloxacin and azithromycin were dissolved to 25 mg/mL using 3.5 µl of glacial acetic acid added to 996.5 µL of sterile 1× phosphate-buffered saline (PBS) and vigorous vortexing. Gentamicin and ceftriaxone were dissolved in 1× PBS to 50 mg/mL stock concentrations.

Standard agar plates were made using regular-strength Gonococcal (GC) agar (BD diagnostics), autoclaved and cooled to 55°C, and supplemented with 1% defined growth supplement (File S1). After serial diluting 20× concentrated antimicrobials in sterile 1× PBS, 1 mL of antimicrobial was added to each petri dish. To achieve a 1× antimicrobial concentration, 19 mL of GC agar was added and adequately mixed before leaving to set at room temperature overnight. The plates were then stored at 4°C for a maximum of 2 weeks.

### 96-well microtiter dilution antimicrobial assay

Two sets of 96-well microtiter assay plates were prepared, and one was supplemented with the complex-defined growth supplement and the other with Kellogg’s (S1). After serial diluting 20× concentrated antimicrobials in sterile 1× PBS, 10 µL of each dilution was added to the first 10 wells of each row. Autoclaved and cooled to 55°C, 190 µL of 1% supplemented GC agar was added and mixed gently to avoid generating bubbles within the wells. Positive and negative growth controls were included by adding 200 µL of agar to the final two wells. Plates were left to set at room temperature overnight and stored at 4°C for 2 weeks. A standardized protocol is available in the Supplemental material.

### Inoculation, incubation, and MIC interpretation

As per CLSI recommendations, inocula grown on chocolate agar for 18–24 hours were prepared in sterile 1× PBS to a 0.5 McFarland Standard. This suspension was diluted 1:9, and then 1 µL of inoculum was applied to the prewarmed plates at 37°C. Quality assurance on the final diluted inocula was performed to confirm the proper CFU/mL was achieved (expected CFU/mL per 1 µL is approximately 1 × 10^4^ CFU/mL). Inoculations were completed in duplicate for each experiment. Plates were incubated in 5% CO2 at 37°C with the lids ajar until drops were absorbed. Once absorbed, plates were incubated for 20–24 hours before reading. Results were observed by the unaided eye, with the MIC being recorded as the first dilution of antimicrobial where no growth was observed. Single colonies and haze of growth were considered negative. The result interpretation was based on CLSI interpretation criteria, except for gentamicin. Because no breakpoint interpretation exists for this organism-antimicrobial combination, breakpoint analysis is suggested by several research groups as follows: ≥4 µg/mL as sensitive, 8–16 µg/mL as intermediate, and ≥32 µg/mL as resistant (17, 29, 30).

### Statistical analysis

Overall, MIC values of the reference agar dilution method were taken as the mode of all replicates per strain, with the more resistant MIC taken in the event of a tie between two dilutions. Replicate values and calculations are reported in Supplemental data. To calculate categorical agreement (CA) for each 96-well microtiter method compared to the reference method, the total number of replicates across the 96-well method with the same breakpoint category as the reference was divided by the total number of replicates tested in the 96-well method (21, 31). Essential agreement (EA) of the 96-well method to the reference was calculated by dividing the total number of replicates within plus or minus 1 dilution factor of the reference method MIC by the total number of replicates tested in the 96-well method (21, 31–33). Errors within the technique were also determined. A major error is defined as a deviation of two categorical breakpoints, while a minor error is defined as a deviation of one categorical breakpoint. Minor errors were calculated by dividing the total number of replicates in the 96-well method that fell outside the breakpoint category determined by the reference by the total number of replicates tested in the 96-well method. To assess the accuracy of categorical assignment and MIC determination, CA and EA should each be ≥90% (31–33). The agar dilution experiment was repeated with a minimum of three biological replicates across a minimum of three technical replicates, and each microtiter experiment was repeated with a minimum of five biological replicates across ten technical replicates.

## RESULTS

To evaluate the performance of the 96-well microtiter assay for *Neisseria gonorrhoeae* antimicrobial susceptibility testing, we compared MIC values generated by the gold standard agar dilution method with those from two 96-well formats: one supplemented with a complex-defined growth supplement and the other with Kellogg’s supplement (Table 1). Seventeen laboratory strains with varying susceptibility profiles were tested against four antimicrobials: azithromycin, ceftriaxone, ciprofloxacin, and gentamicin. Each antimicrobial strain-method combination was assessed in multiple biological and technical replicates.

**TABLE 1:** Minimum inhibitory concentrations (µg/mL) of *N. gonorrhoeae* determined using agar dilution, 96-microtiter assay supplemented with either complex-defined supplement or supplemented with Kellogg’s supplement^*b*^

	Antimicrobial^*a*^											
	Azithromycin			Ceftriaxone			Ciprofloxacin			Gentamicin		
Strain	A	96-I	96-K	A	96-I	96-K	A	96-I	96-K	A	96-I	96-K
49226	0.25	0.5	0.25	0.0156	0.0156	0.0156	0.002	0.002	0.0039	4	8	8
FA1090	0.0625	0.125	0.125	0.0039	0.002	0.0039	0.0039	0.0039	0.0039	16	8	8
F62	0.0625	0.125	0.0625	0.001	0.001	0.001	0.002	0.002	0.0039	16	16	8
CH811	0.125	0.125	0.125	0.0039	0.0039	0.0039	0.002	0.002	0.0039	8	16	8
WHO F	0.25	0.125	0.25	<0.001	<0.001	<0.001	0.0039	0.0039	<0.001	8	8	8
WHO G	0.5	0.25	0.25	0.0156	0.0156	0.0156	0.125	0.125	0.125	8	8	8
WHO K	0.5	0.5	0.25	0.25	0.25	0.125	32	32	32	8	8	8
WHO L	0.5	0.25	0.5	0.25	0.125	0.125	32	32	16	8	4	8
WHO M	1	1	0.5	0.031	0.031	0.031	2	2	2	8	16	16
WHO N	0.25	0.25	0.25	0.0078	0.0078	0.0078	4	8	8	8	8	16
WHO O	0.25	0.5	0.5	0.031	0.0156	0.031	0.0156	0.0078	0.0078	8	8	8
WHO P	4	4	4	0.0156	0.0156	0.0156	0.0078	0.0156	0.0078	16	8	8
WHO V	≥512	≥512	512	0.0625	0.031	0.031	32	32	32	32	32	16
WHO W	0.5	0.25	0.25	0.125	0.0625	0.125	32	32	32	16	8	16
WHO X	0.5	0.5	0.5	2	2	2	64	64	64	16	8	16
WHO Y	0.5	0.5	0.5	2	2	2	32	16	16	16	8	16
WHO Z	1	0.5	0.5	1	1	0.5	32	32	32	16	16	8

^*a*^ MICs were determined for azithromycin, ceftriaxone, ciprofloxacin, and gentamicin. Plates were inoculated with standardized *N. gonorrhoeae* inoculum and incubated at 37°C with 5% CO₂ and 95% humidity for 20–24 hours. MIC observations were made by eye; single colonies and haze of growth were considered negative. MIC results are reported as the mode result of 3–5 replicates for the agar dilution and 6–10 replicates in the 96-well microtiter assay.

^*b*^ A=agar dilution, 96-I=96-well microtiter with Isovitalex supplement, 96-K=96-well microtiter with Kellogg’s.

Across all antimicrobials and methods, no major errors were observed. Minor errors were rare among azithromycin, ceftriaxone, and ciprofloxacin assays, with most strain method combinations showing 0% minor error rates. A single exception occurred with strain WHO P tested against azithromycin using Kellogg’s supplement, where a minor error rate of 14% was observed (Table 2). By contrast, gentamicin exhibited substantial variability, with minor error rates ranging from 0% to 100% depending on the strain and testing condition (Tables 2 and 3).

**TABLE 2:** Comparison of categorical agreement, essential agreement, and minor error rates between agar dilution and 96-well microtiter supplemented with complex-defined supplement

	Antimicrobial^*a*^											
	Azithromycin			Ceftriaxone			Ciprofloxacin			Gentamicin		
	CA	EA	ME	CA	EA	ME	CA	EA	ME	CA	EA	ME
Strain												
49226	100	100	0	100	100	0	100	100	0	33	100	67
FA1090	100	100	0	100	100	0	100	67	0	83	83	17
F62	100	100	0	100	100	0	100	100	0	67	67	33
CH811	100	100	0	100	100	0	100	100	0	100	100	0
WHO F	100	100	0	100	100	0	100	100	0	100	100	0
WHO G	100	100	0	100	100	0	100	100	0	100	100	0
WHO K	100	100	0	100	83	0	100	100	0	100	100	0
WHO L	100	100	0	100	100	0	100	100	0	33	100	67
WHO M	100	100	0	100	100	0	100	100	0	67	100	33
WHO N	100	100	0	100	100	0	100	100	0	100	100	0
WHO O	100	100	0	100	100	0	100	100	0	100	100	0
WHO P	100	100	0	100	100	0	100	100	0	75	75	25
WHO V	100	100	0	100	100	0	100	100	0	33	67	67
WHO W	100	100	0	100	100	0	100	100	0	100	100	0
WHO X	100	100	0	100	100	0	100	83	0	100	100	0
WHO Y	100	67	0	100	100	0	100	100	0	100	100	0
WHO Z	100	33	0	100	100	0	100	100	0	67	67	33

^*a*^ Results are shown for the 96-well microtiter assay supplemented with complex-defined growth supplement. Categorical agreement (CA) represents the percentage of replicates in the same sensitivity category as the reference method. Essential agreement (EA) indicates the percentage of replicates within ±1 dilution of the reference method. Minor error (ME) represents the percentage of replicates falling outside the reference category, within one category.

**TABLE 3:** Comparison of categorical agreement, essential agreement, and minor error rates between agar dilution and 96-well microtiter supplemented with Kellogg’s supplement

	Antimicrobial^*a*^											
	Azithromycin			Ceftriaxone			Ciprofloxacin			Gentamicin		
	CA	EA	ME	CA	EA	ME	CA	EA	ME	CA	EA	ME
Strain												
49226	100	100	0	100	100	0	100	100	0	0	100	100
FA1090	100	100	0	100	100	0	100	67	0	100	100	0
F62	100	100	0	100	100	0	100	100	0	100	100	0
CH811	100	100	0	100	100	0	100	100	0	100	100	0
WHO F	100	100	0	100	100	0	100	33	0	50	75	4
WHO G	100	100	0	100	100	0	100	100	0	75	100	25
WHO K	100	100	0	100	100	0	100	100	0	75	100	25
WHO L	100	86	0	100	67	0	100	100	0	100	100	0
WHO M	100	100	0	100	100	0	100	100	0	100	100	0
WHO N	100	100	0	100	100	0	100	100	0	100	100	0
WHO O	100	100	0	100	67	0	100	71	0	88	100	13
WHO P	86	86	14	100	100	0	100	100	0	100	100	0
WHO V	100	100	0	100	100	0	100	100	0	25	100	75
WHO W	100	86	0	100	100	0	100	100	0	100	100	0
WHO X	100	86	0	100	100	0	100	100	0	100	100	0
WHO Y	100	83	0	100	100	0	100	83	0	100	100	0
WHO Z	100	67	0	100	100	0	100	100	0	100	100	0

^*a*^ Results are shown for the 96-well microtiter assay supplemented with Kellogg’s growth supplement. Categorical agreement (CA) represents the percentage of replicates in the same sensitivity category as the reference method. Essential agreement (EA) indicates the percentage of replicates within ±1 dilution of the reference method. Minor error (ME) represents the percentage of replicates falling outside the reference category, within one category.

Categorical agreement (CA) and essential agreement (EA) were calculated relative to agar dilution results. For the complex-defined growth supplement microtiter assay, CA was 100% for azithromycin, ceftriaxone, and ciprofloxacin across all tested strains. For gentamicin, CA ranged from 33% to 100%, depending on the strain. EA values for this method spanned 33% to 100%, again with gentamicin showing the lowest agreement (Table 2).

For Kellogg’s supplement microtiter assay, CA for azithromycin, ceftriaxone, and ciprofloxacin ranged from 88% to 100%. Gentamicin CA varied widely, from 0% to 100%. EA values for this format ranged from 25% to 100% across antimicrobials, with azithromycin, ceftriaxone, and ciprofloxacin showing consistently high agreement and gentamicin again demonstrating the greatest variability (Table 3).

**Fig 2:**
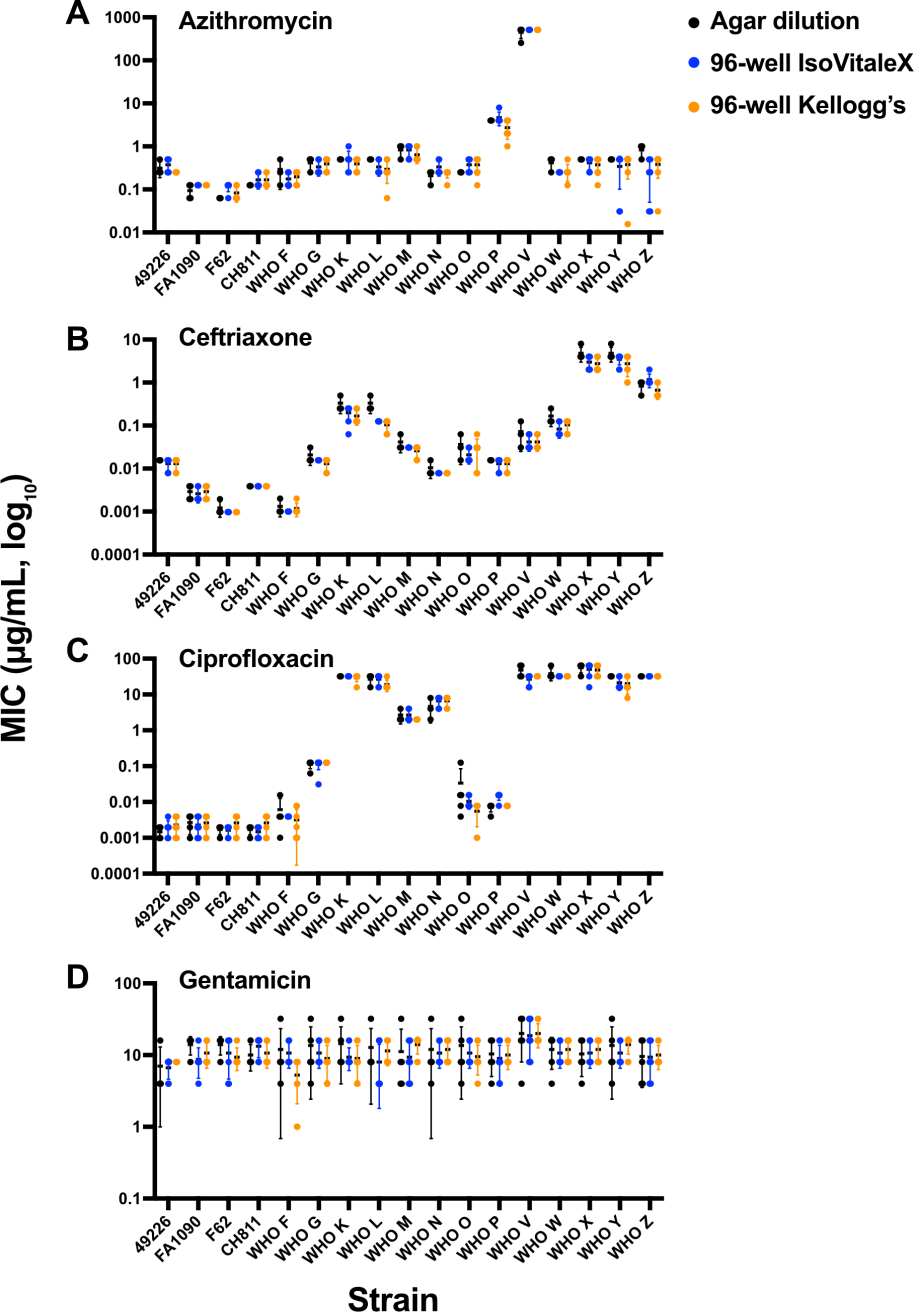
Comparison of MICs of *N. gonorrhoeae* strains tested using agar dilution and 96-well microtiter assays. MICs for (A) azithromycin, (B) ceftriaxone, (C) ciprofloxacin, and (D) gentamicin were determined using three methods: agar dilution (black), 96-well microtiter assay supplemented with complex-defined growth supplement (IsoVitaleX) (blue), and 96-well microtiter assay supplemented with Kellogg’s supplement (orange). A panel of 17 *N*. *gonorrhoeae* strains, including WHO reference strains, was tested. MIC values are shown on a log₁₀ scale. Each point represents a biological replicate; bars indicate the median and range.

To visualize the distribution of MIC values across all testing methods, individual replicate results for each strain were plotted by antimicrobial (Fig. 2). For azithromycin, ceftriaxone, and ciprofloxacin, MIC values were tightly clustered within strains and showed strong concordance between agar dilution and both 96-well microtiter formats. By contrast, gentamicin MICs exhibited broader variability both within and across methods.

**Fig 3:**
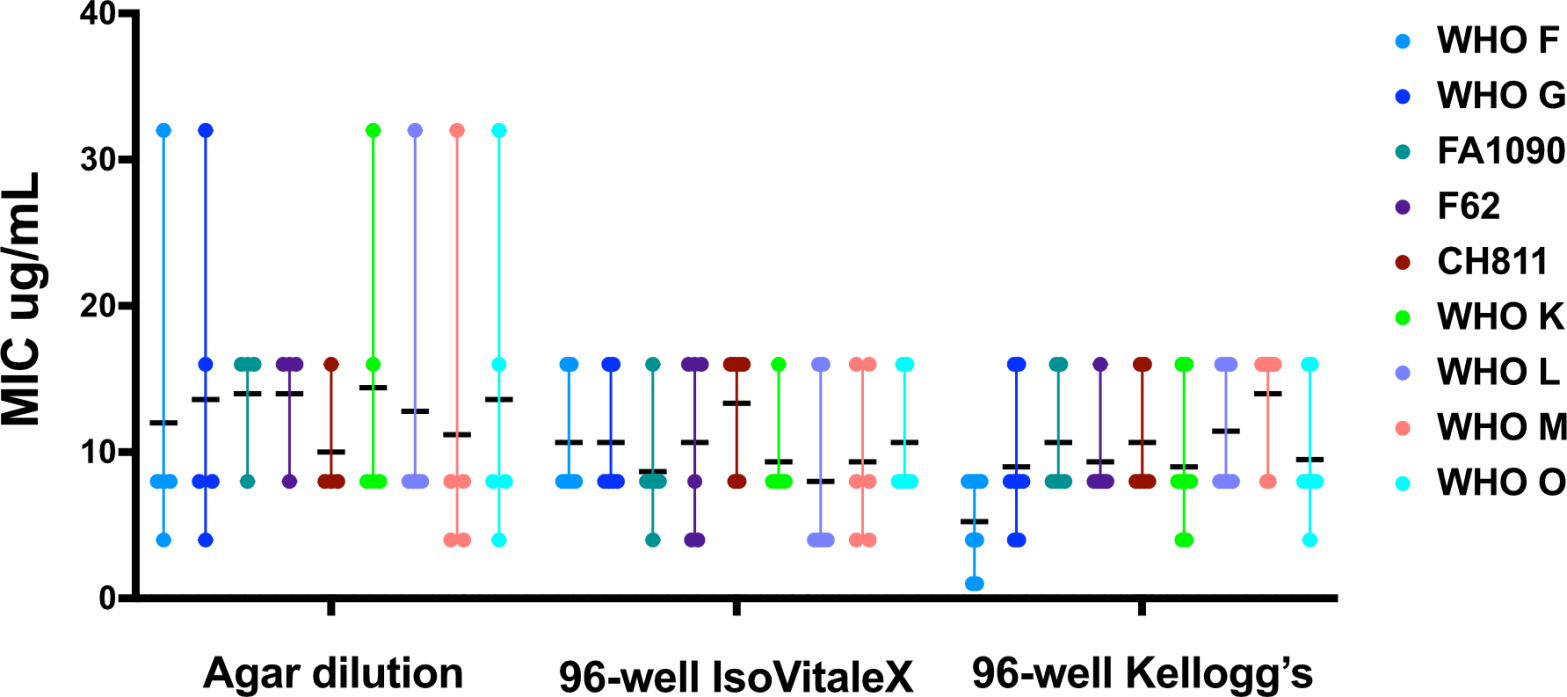
Gentamicin MICs for selected *N. gonorrhoeae* strains. Strains were chosen based on variability in gentamicin MICs observed across methods. Each colored dot represents a biological replicate; horizontal black bars indicate median MIC values. MICs are displayed on a linear scale. Strain colors are consistent across all conditions to highlight method-dependent variation.

To further explore this observation, a subset of strains with the most pronounced gentamicin variability was selected for detailed analysis (Fig. 3). This included reference and laboratory strains with greater inter-replicate spread or method-dependent MIC shifts. As shown in Fig. 3, variation in gentamicin MICs was not attributable to a single method; instead, several strains demonstrated fluctuation in MICs across agar dilution and 96-well formats. In some cases, replicate values for a single strain spanned more than one interpretive category (e.g., susceptible vs. intermediate), underscoring the need for multiple replicates and cautious interpretation of gentamicin susceptibility in *N. gonorrhoeae*. These findings highlight gentamicin as an antimicrobial replicating variability and proximity to breakpoint thresholds, which may complicate categorical interpretation.

## DISCUSSION

Antimicrobial sensitivity testing for *N. gonorrhoeae* requires a specialized approach distinct from that used for non-fastidious pathogens. Reliable access to the reference method is critical for laboratories involved in the study and surveillance of this organism. We considered two key perspectives to validate our method: clinical and research applications. For clinical-focused applications, the focus lies on categorical breakpoints, with CA values and major/minor error rates serving as benchmarks for assay validation. For research laboratories, precision in measuring MICs is paramount, making EA a critical metric for experimental validation. Our results demonstrate that the 96-well microtiter assay meets the criteria for both applications. Furthermore, we validated the assay with Kellogg’s supplement, providing a simpler alternative without compromising accuracy.

The use of a 96-well microtiter platform using either complex-defined or Kellogg’s supplements for susceptibility testing of azithromycin, ciprofloxacin, and ceftriaxone performs comparably to the traditional agar dilution method. Our results demonstrate CA and minor error rates to be within acceptable limits. However, our EA demonstrates there is variability between biological replicates when using the 96-well microtiter assay. The variability observed highlights the importance of applying quality control measures and biological replicates to ensure the reliability of the results obtained. Notably, there was no consistency between EA rates being below 90% for any specific strain versus antimicrobial, with the 96-well Kellogg’s supplemented assay showing a higher EA range. The variability observed may be attributed to several factors, such as technical proficiency when preparing and inoculating the plates, individual interpretation biases (such as what is considered a haze of growth versus light growth), and/or the inherent variability naturally expected in susceptibility testing. With gentamicin, CA, EA, and minor error rates demonstrated high rates of variability. One consideration that should be made is that the MIC results of our strains tested all consistently fell around the threshold of sensitive (≤4 µg/mL) and intermediate (8–16 µg/mL). This is likely the greatest contributing factor, along with the above-stated factors. Minor variations in MICs are common with repeated testing, particularly for isolates with MICs near the thresholds of two categories (e.g., sensitive and intermediate) (17). Despite this variability, the use of a 96-well microtiter assay supplemented with either complex-defined or Kellogg’s supplement is comparable to the gold standard method, provided appropriate experimental replicates and quality control measures are used to ensure the precision of the method. Due to the technical nature of preparing the plates and inocula, considerations should be made if aspects of the testing process can be automated (such as the use of robotics) to reduce human error. Overall, this dual-purpose assay offers an accessible and efficient platform for different research applications, advancing antimicrobial resistance surveillance and supporting experimental workflows.

Over the past decade, the detection of *N. gonorrhoeae* has shifted from culture-based methods to molecular-based approaches, with follow-up antimicrobial susceptibility testing restricted to a subset of clinical cases meeting specific criteria. However, *N. gonorrhoeae* is historically known for its rapid acquisition of resistance to therapeutic antimicrobials (34, 35). Globally, isolates have exhibited increasing MICs for key antimicrobials such as azithromycin and ceftriaxone, with a growing number of strains displaying resistance (36–39). The World Health Organization’s Gonococcal Antimicrobial Surveillance Programme (GASP) collects global surveillance data to inform treatment guidelines for gonorrhea (40). According to WHO recommendations, empiric use of an antimicrobial should be discontinued if resistance or treatment failure rates exceed 5% (41). This highlights the need to reevaluate the frequency of antimicrobial susceptibility testing in surveillance practices, ensuring preparedness for an anticipated increase in culture and testing of this fastidious pathogen.

The use of agar microdilution for testing antimicrobial compounds has been previously demonstrated with non-fastidious organisms such as *Escherichia coli* and *Pseudomonas aeruginosa* in 35 mm Petri dishes (42). Golus et al. later adapted this technique to a 96-well microdilution assay (43). Our study builds on these methods, applying the 96-well microtiter assay to *N. gonorrhoeae* susceptibility testing. The assay exhibited strong replicability compared to the gold standard agar dilution method. From a clinical perspective, no major errors were observed across antimicrobials when using the microdilution assay with Kellogg’s supplement, supporting its validity as an alternative method for assessing *N. gonorrhoeae* susceptibility. From a research perspective, the assay demonstrated consistent EA across strains, with some variability, making it a reliable platform for studying antimicrobial resistance and screening novel compounds.

AMR research has seen significant advancements over the past decade, focusing on molecular assays such as nucleic acid amplification tests (NAATs) and whole-genome sequencing to identify resistance determinants (44–46). Molecular assays for the detection of specific AMR determinants have started to be utilized in clinical diagnostics to improve therapeutic decision-making (47). More promising research suggests that multiplex PCR assays can predict susceptibility to azithromycin, cephalosporins, and ciprofloxacin in NAAT-positive samples with high accuracy (48). These assays, which do not rely on viable organisms, are critical for large-scale surveillance programs and detecting multiple AMR determinants in a single test, particularly as most clinical laboratories lack in-house culture and susceptibility testing capabilities. Despite these advancements, culture-based methods remain indispensable for monitoring *N. gonorrhoeae* resistance. They are essential for detecting resistance mechanisms that may not yet be associated with known molecular markers (44, 45). Furthermore, the pathogen’s phase and antigenic variation can result in phenotypes that deviate from genotypic predictions, complicating molecular diagnostics (49–51).

To this end, our study demonstrates that agar dilution can be effectively scaled down to a manageable 96-well microtiter assay, increasing the accessibility of this reference method. This platform not only supports the development of rapid molecular assays but also facilitates the testing of novel antimicrobial compounds by streamlining experimental workflows. However, limitations in this study include the small number of laboratory strains tested and the limited antimicrobials tested using this platform. Future validation efforts should incorporate a broader range of clinical isolates and antimicrobials to strengthen the assay’s robustness. In addition, exploring automation of the assay could further reduce the time required for media preparation and inoculation, reducing technical errors.

### Conclusion

As the need for comprehensive antimicrobial surveillance of *N. gonorrhoeae* grows, our study demonstrates that the 96-well microtiter dilution assay is a viable alternative to the gold standard agar dilution method for testing azithromycin, ceftriaxone, gentamicin, and ciprofloxacin. This approach offers significant advantages, including reduced labor and resource requirements, making it more accessible for research laboratories. Moreover, its scalability enables high-throughput screening applications, such as screening for novel antimicrobial compounds and rapidly validating new molecular AMR testing methods. By increasing the efficiency and accessibility of the gold standard susceptibility testing, this assay can strengthen global efforts in monitoring and combating antimicrobial resistance in *N. gonorrhoeae*.

## SUPPLEMENTAL MATERIAL

Supplemental material spectrum.03249-24-s0001.docx MIC protocols.

Supplemental data spectrum.03249-24-s0002.xlsx MIC validation calculations.

ASM does not own the copyrights to Supplemental Material that may be linked to, or accessed through, an article. The authors have granted ASM a non-exclusive, world-wide license to publish the Supplemental Material files. Please contact the corresponding author directly for reuse.
