# Supplementary material for: Neisseria gonorrhoeae antimicrobial susceptibility testing: application of traditional agar dilution to a high-throughput 96-well microtiter assay
Source: Microbiol Spectr. 2025 Jun 9;13(7):e03249-24. doi: 10.1128/spectrum.03249-24 (PMC12210901; doi:10.1128/spectrum.03249-24)
Supplement: Supplemental material — MIC protocols. [file spectrum.03249-24-s0001.docx]

**SUPPLEMENTAL INFORMATION**

***Neisseria gonorrhoeae* antimicrobial susceptibility testing: application of traditional agar dilution to a high throughput 96-well microtiter assay**

Joyce E. Kuipers, Laura Seidel, Nicole E. Garnier and Cezar M. Khursigara*

Department of Molecular and Cellular Biology, College of Biological Science,

University of Guelph, N1G 2W1 Guelph, Ontario, Canada.

**Supplements and media**

**1% defined growth supplement (as per CLSI M100):**

For a total volume of 1.0 L in ddH2O:

-1.1 g L-cystine -0.03 g guanine HCl

-0.003 g thiamine HCl -0.013 g para-aminobenzoic acid

-0.01 g B12 -0.1 g co-carboxylase (TTP)

-0.25 g NAD -1 g adenine

-10 g L-glutamine -100 g glucose

-0.02 g ferric nitrate -25.9 g L-cysteine HCl

Add ingredients one at a time, mix thoroughly and sterilize using a 0.22 um filter. Store at 2-8ºC for up to 4 weeks. Alternatively, the addition of small volumes of 1 molar HCl minimizes precipitation of components, allowing for freezing. This extends the lifespan of the supplement, however, ensure to monitor the quality of the supplement over time. Thaw at room temperature. Avoid refreezing.

**Kellogg’s supplement:**

For a total volume of 100 mL in ddH2O:

-40 g glucose -1 g glutamine

-10 mL 0.5% ferric nitrate -200 mg co-carboxylase (TTP)

Fill flask with 100 mL of ddH2O, mark the line and remove about 50 mL. Over low heat, add glucose and glutamine and mix until dissolved. Add 200 mg of TTP and mix until dissolved. Remove from heat and add 10 mL of 0.5% ferric nitrate solution. Top the flask up to 100 mL, sterilize with a 0.22 um filter.

Store at 2-8ºC for up to 4 weeks to ensure components do not precipitate out of solution.

**Gonococcal base media agar:**

To 500 mL of ddH2O:

-18 g GC base media

Mix until dissolved. Autoclave using a 30-minute sterilization period, cool to 55ºC in a water bath. Once cooled, add 5 mL of Kellogg’s supplement, mix well, and pour approximately 25 mL of media if preparing 100 mm petri dishes or 20 mL per 96-well petri dish. Let set at room temperature overnight, store between 2-8ºC for up to 4 weeks.

**Chocolate agar:**

In separate flasks:

To 250 mL of ddH2O:

-18 g GC base media

To 250 mL of ddH2O:

-5 g hemoglobin

Mix each component until dissolved. Autoclave separate flasks using a 30-minute sterilization period, cool to 55ºC in a water bath. Once cooled, combine flasks and add 5 mL of Kellogg’s supplement. Mix well and pour approximately 25 mL into 100 mm petri dishes. Let set at room temperature overnight, store between 2-8ºC for up to 4 weeks.

**96-well microtiter assay preparation, inoculum and interpretation**

**96-well microtiter agar dilution assay:**

1. Antimicrobial preparation in 96-well plate:
   1. Determine antimicrobial or other agent of interest and concentration range over 10 dilutions. Prepare a stock solution at 40X the highest concentration to be tested. Filter sterilize with 0.22 µm filter.
   2. Add 100 µL 1X PBS to 10 wells of a 96-well plate. Add 100 µL of the 40X stock to the first well, then serially dilute agent of interest across the remaining wells. This results in 100 µL of 20X concentration per well.
2. Agar preparation:
   1. Add 18 g of Gonococcal (GC) base media to 500 mL of dH_2_O. Mix well and autoclave for a 30-minute sterilization cycle.
   2. Cool agar down to 55ºC in a water bath. Once cooled, add 5 mL of supplement, and return to the water bath until ready for use.
3. 96-well microtiter plate preparation:
   1. Using a multichannel pipette, transfer 10 µL of the 20X working concentration of agent of interest from highest concentration (well 1) to lowest concentration (well 10). Wells 11 and 12 are reserved for the positive and negative growth controls, respectively.
   2. Once GC agar is ready, mix thoroughly and transfer the media to a reservoir. Using a multichannel pipette, aspirate 190 µL of media.
   3. In wells 1-10, dispense the 190 µL of media to the first stop of the pipette, avoiding the second stop to prevent the generation of bubbles. Pipette gently up and down 3-5 times to ensure proper mixing, again avoiding the generation of bubbles.
   4. Add 200 µL of media to the positive and negative control wells.
   5. Let plates set for 4 hours at room temperature, then transfer to storage at 2-8ºC for a maximum of 2 weeks.

**Inoculation, incubation, and result interpretation**

1. To prepare inoculums for testing:
   1. Streak isolates from -80ºC storage to non-selective, non-differential media (such as chocolate agar) prewarmed to 37ºC.
   2. Grow for 18-24 hours at 37ºC in 5% CO_2_  and 95% humidity.
   3. Passage isolates to non-selective, non-differential prewarmed media such as chocolate agar or GC base agar. Grow for 18-24 hours in the above-mentioned conditions.
2. For a minimum of 30 minutes before inoculation, place prepared 96-well agar dilution plates in a dry, 37ºC incubator.
3. To prepare inoculum, continuing from step 1.3:
   1. Using sterile isotonic solution such as 1X PBS or 0.9% sodium chloride, prepare a 0.5 McFarland Standard by taking 4-5 isolated colonies. Use the standard within 15 minutes to avoid loss of viable cells.
   2. Dilute 0.5 McFarland Standard 1:9 using the same sterile isotonic solution.
4. Add 1 µL of prepared inoculum to each well, excluding the negative control well.
5. Allow drops to dry at 37ºC and 5% CO_2_ with the lids ajar. Once absorbed, incubate for 20-24 hours.
6. To interpret plates:

6.1 Assess positive and negative control wells for growth and no growth, respectively, to ensure validity of the assay.

6.2 MIC is the first well that no growth is observed. Hazy growth or a single colony are considered a negative result.
